# Behavioral domains in compulsive rats: implications for understanding compulsive spectrum disorders

**DOI:** 10.3389/fnbeh.2023.1175137

**Published:** 2023-05-18

**Authors:** Elena Martín-González, Manuela Olmedo-Córdoba, Ángeles Prados-Pardo, Daniel J. Cruz-Garzón, Pilar Flores, Santiago Mora, Margarita Moreno-Montoya

**Affiliations:** ^1^Department of Psychology and Health Research Centre (CEINSA), University of Almería, Almería, Spain; ^2^Department of Neuroscience and Panum Institute, University of Copenhagen, Copenhagen, Denmark

**Keywords:** compulsive behavior [F01-145-527-100], schedule-induced polydipsia, cognitive control system, negative valence system, risky decision-making, cognitive impulsivity, behavioral flexibility, Research Domain Criteria (RDoC)

## Abstract

**Introduction:**

Compulsive behavior has been proposed as a transdiagnostic trait observed in different neuropsychiatric disorders, such as obsessive-compulsive disorder, autism, and schizophrenia. Research Domain Criteria (RDoC) strategy could help to disentangle the neuropsychological basis of compulsivity for developing new therapeutic and preventive approaches. In preclinical research, the selection of high-drinker (HD) vs. low-drinker (LD) animals by schedule-induced polydipsia (SIP) is considered a putative model of compulsivity, which includes a well-differentiated behavioral pattern.

**Methods:**

The purpose of this research was to assess the cognitive control and the negative valence system domains in a phenotype of compulsive HD rats. After the selection of animals as HD or LD, we assessed behavioral inflexibility by probabilistic spatial reversal learning (PSRL), motor and cognitive impulsivity by variable delay-to-signal (VDS), and risky decision-making by rodent gambling task (rGT).

**Results:**

HD rats performed fewer reversals and showed less probability of pressing the same lever that was previously reinforced on PSRL, more premature responses after the exposure to longer delays on VDS, and more disadvantageous risky choices on rGT. Moreover, HD animals performed more perseverative responses under the punishment period on rGT.

**Discussion:**

These results highlight that HD compulsive phenotype exhibits behavioral inflexibility, insensitivity to positive feedback, waiting impulsivity, risky decision-making, and frustrative non-reward responsiveness. Moreover, these findings demonstrate the importance of mapping different behavioral domains to prevent, treat, and diagnose compulsive spectrum disorders correctly.

## 1. Introduction

Compulsivity can be defined as a perseveration of a response that is irresistible and inappropriate to the individual and unavoidable despite its negative consequences (Robbins and Crockett, 2010). The presence of compulsions, which are stereotyped behaviors conducted following rigid rules and performed to decrease or avoid unpleasant consequences (Chamberlain et al., 2009), is the core feature observed in obsessive-compulsive disorder (OCD), which affects between 1.1 and 1.8% of the population internationally (American Psychiatric Association, 2013). Nowadays, compulsivity could be considered a transdiagnostic trait, which may be a problem for traditional diagnostic systems, prevention, and treatment (Den Ouden et al., 2020). In this sense, as a result of neuroscience insights (for a review, see Fineberg et al., 2018), the diagnostic classification systems DSM-5 (American Psychiatric Association, 2013) and ICD-11 (World Health Organization, 2018) have removed OCD from the anxiety disorder grouping, and it now stands at the head of a new family of obsessive-compulsive spectrum disorders (otherwise known as obsessive-compulsive and related disorders, OCRDs), including body dysmorphia, hoarding, hair-pulling, skin picking and olfactory reference disorders, and hypochondriasis, all sharing compulsive behavior as a cardinal characteristic (Fineberg et al., 2020). The Roadmap for Mental Health Research in Europe (ROAMER) (Haro et al., 2014) and the Research Domain Criteria (RDoC) by the U.S. National Institute of Mental Health (Insel et al., 2010) are new research strategies based on the dimension of altered behavior (Fineberg et al., 2016). There is a growing interest in the RDoC initiative, highlighting the importance of identifying the behavioral and cognitive domains related to compulsive behavior. However, this characterization has been mainly based on cognitive domains, excluding other important information related to emotional processing, which could lead to a better characterization of possible endophenotypes (Moreno-Montoya et al., 2022).

Growing evidence suggests the existence of different cognitive mechanisms mediating compulsive behavior in a broad range of compulsive disorders (for a review, see Fineberg et al., 2014). These theories have included behavioral inhibition, cognitive flexibility, and decision-making deficits. Regarding behavioral inhibition, impulsive behavior is defined as a tendency to act prematurely without foresight and involves actions that are insufficiently conceived, prematurely expressed, excessively risky, or inappropriate to the situation (Dalley et al., 2011). It is a non-unitary phenomenon that might be observed in two forms: motor impulsivity, understood as excessive behavior, and cognitive impulsivity, determined by choice (Chudasama et al., 2003; Winstanley et al., 2004). Several clinical studies suggest that impulsivity may be a feature of OCD (Ettelt et al., 2007; Benatti et al., 2014). Related to cognitive impulsivity, risky decision-making has been proposed recently as a core dimension of OCD (Grassi et al., 2015, 2018, 2020). Moreover, cognitive flexibility can be defined simply as “adjusting to change” and involves the ability to switch or shift from thinking about one conceptual representation to another, especially in response to changes in rules and environmental feedback (Chamberlain et al., 2021). Cognitive flexibility impairment has been observed in OCD patients and their unaffected relatives (Chamberlain, 2006; Chamberlain et al., 2007; Patel et al., 2010; Voon et al., 2015; Vaghi et al., 2017).

Interestingly, the deficit in the expression of cognitive flexibility and risky decision-making tasks in the compulsive phenotype might reflect an aberrant processing of the consequences, once learning has occurred, linked to emotional domains. Thus, reward processing during a compulsion or after avoiding an undesired consequence might also be critical in the maintenance of compulsive behavior. In fact, a dysfunctional reward circuit has been proposed in OCD and gambling disorder (GD) patients (Grassi et al., 2020). OCRD patients might engage in repetitive and rigid behaviors as the development of a dependency over time upon their compulsions due to the rewarding effect when performed perfectly or when compulsions reduce obsession-induced distress (Denys, 2011). This evidence points out that altered cognitive control and negative valence system domains might naturally overlap in the compulsive phenotype.

Preclinical models allow us to provide a clearer understanding of the behavioral processes underpinning the compulsive phenotype. In this sense, the state of food deprivation induces the acquisition and expression of adjunctive behavior under different fixed time (FT) or fixed interval (FI) schedules (Falk, 1966, 1971; López-Crespo et al., 2004). Schedule-induced polydipsia (SIP) is one of the most well-established preclinical models for the study of neuropsychopathological disorders presenting compulsive behavior. After 15–20 sessions of SIP procedure, important individual differences in drinking behavior have been observed, and animals can be divided by the median split into two different populations: high drinker (HD) considered as high compulsive phenotype and low drinker (LD) considered as non-compulsive rats(Cardona et al., 2006, 2011; López-Grancha et al., 2006, 2008; Moreno et al., 2010; Pellón et al., 2011; Moreno and Flores, 2012; Navarro et al., 2015, 2017; Merchán et al., 2017, 2019; Martín-González et al., 2018, 2022b; Mora et al., 2018, 2020; Prados-Pardo et al., 2019), which follows a bimodal distribution (Mora et al., 2018). According to the criteria for assessing animal models (Markou et al., 2009), SIP fulfills the face, construct, and predictive validity criteria of a preclinical model of compulsive behavior (for a review, see Moreno and Flores, 2012). In this sense, drinking behavior on SIP is (1) excessive, because the amount of water ingested reaches in some cases one-half of their body weight in water (Falk, 1966); (2) persistent across the session and despite negative consequences such as when water is substituted by quinine (Fouyssac et al., 2021); and (3) maladaptive behavioral habit, as it is not a result of physiological needs (Falk, 1961, 1966, 1971). Thus, SIP preclinical model allows us to identify a compulsive vulnerable population to study behavioral (Moreno-Montoya et al., 2022) and neurochemical alterations (Martín-González et al., 2022a) to extend our knowledge of common compulsive spectrum disorders due to their transdiagnostic profile as, for example, in addiction, schizophrenia, or OCD (Moreno and Flores, 2012; Belin-Rauscent et al., 2016; Navarro et al., 2017).

For this purpose, this study aimed to further characterize the compulsive phenotype selected by SIP, assessing different RDoC domains related to the cognitive control system and the negative valence system, such as motor inhibition by variable delay-to-signal (VDS) task, cognitive impulsivity by VDS, behavioral inflexibility by probabilistic spatial reversal learning (PSRL) task, risky decision-making by rodent gambling task (rGT), and frustrative non-reward by rGT. The identification of behavioral, cognitive, and emotional aspects altered in compulsive selected animals could help in developing evidence-based strategies for the diagnosis of different compulsive profiles to better prevent and treat psychopathological disorders related to compulsivity.

## 2. Material and methods

### 2.1. Subjects

In total, 48 male Wistar rats from Envigo (Barcelona, Spain), weighing between 225 and 250 g at the beginning of the experiment, were used in the present study. The animals were housed four rats per cage (50 × 35 × 20 cm) and kept in a 12:12 h light–dark cycle (lights off at 08:00 h) in a temperature-controlled environment at 22°C. Water and food were freely available, and environmental enrichment, consisting of wooden blocks and PVC tubes, was provided throughout the experiment. After 10 days for habituation and before behavioral tasks, animals through controlled feeding were gradually reduced to 85% of their free-feeding body weight relative to a standard growth curve available at the provider's website. Then, 30 min after each daily experimental session, food was provided. All testing was carried out between 9:00 and 15:00 h. All the procedures were approved by the Committee of Ethics of the University of Almería and by the Junta de Andalucía and were carried out in accordance with the Spanish Royal Decree 53/2013 and the European Community Directive (2010/63/EU) for animal research. This study complied with the ARRIVE guidelines. The authors declare that the research shows commitment to the 3Rs principle (replacement, reduction, refinement). Throughout the entire experiment, adequate measures were taken to minimize pain or discomfort for the experimental animals.

### 2.2. SIP procedure

#### 2.2.1. Description of the apparatus

Rats were tested in eight standard operant chambers (32 × 25 × 34 cm) (MED Associates, St. Albans, VT, USA) equipped with a pellet dispenser, bottle of water, and ambient light. The programming and recording of experimental events were automatically controlled using Med PC IV computer and commercial software (Cibertec SA, Spain).

#### 2.2.2. Behavioral procedure

Before carrying out SIP, for two consecutive days, the amount of water ingested was evaluated for 60 min to obtain a baseline. There was unlimited access to a bottle of fresh water and a reward of 60 pellets (Noyes 45-mg dustless reward pellets; TSE Systems, Germany) deposited together in each feeder in each baseline session. After 1 day of habituation to the operant boxes, rats were exposed during 60-min sessions to a food pellet presentation using a fixed time 60s (FT-60s) schedule. There was a bottle containing fresh tap water on the wall opposite the pellet dispenser. After 20 daily sessions and following the protocol described by Moreno and Flores (2012), animals were classified into low drinkers (LD) and high drinkers (HD), depending on whether the water intake (average of the last five sessions) was above or below the median of the group. The following measures were recorded for each rat: the total amount of water (ml) removed from the bottle, the total number of licks to the bottle, and the total number of entries to the food storage area (Mora et al., 2018).

### 2.3. Experimental design

**Figure 1:**
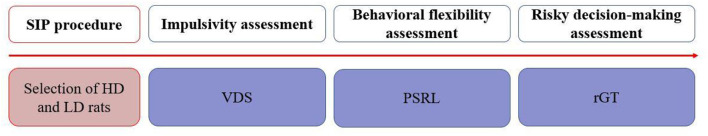
Experimental procedure illustrated in a timetable. HD, high drinker; LD, low drinker; PSRL, probabilistic spatial reversal learning; rGT, rodent gambling task; SIP, schedule-induced polydipsia; VDS, variable delay-to-signal.

The order of behavioral testing was as follows: SIP, impulsivity measures (variable delay-to-signal, VDS), behavioral flexibility measure (probabilistic spatial reversal learning, PSRL), and risky decision-making measures (rodent gambling task, rGT). Each task commenced 20 days after the previous one in order to avoid possible interferences between them, as they could not be randomized due to their different duration in days. The experimental events are summarized in Figure 1.

#### 2.3.1. Variable delay-to-signal: motor and cognitive impulsivity

##### 2.3.1.1. Description of the apparatus

Animals were tested using six standard operant chambers identical to those described in the SIP procedure section with an array of five contiguous square holes opposite the pellet dispenser. These apertures had photocell beams at the entrance and a yellow stimulus light for the nose-poke response. Just the center hole was active in this task.

##### 2.3.1.2. Behavioral procedure

After 15 min of habituation to the test environment with free reward pellets in the pellet dispenser and in the center hole, the protocol for VDS training was initiated. The training sessions started with turning on the house light, delivering one pellet in the pellet dispenser, and the collection of which initiated an intertrial interval (ITI) of 3 s. Next, trials started with 3 s (delay period) with only the house light on followed by the lighting of the center hole for 60 s (response period). A nose poke in this hole was either rewarded with a pellet if performed during the response period or punished with a timeout period in complete darkness (5 s) if performed during the delay period (premature responses). Pellet collection triggered a 3-s ITI before a new trial began. Each training session terminated following 30 min or after 100 trials, whichever occurred first. The training sessions were carried out twice daily, with a 5-h interval in between, for five consecutive days. The VDS experimental session consisted of 120 trials similar to those described in training sessions, except that the delay was 3 s in the first and the last 25 trials and randomly either 6 or 12 s in the middle 70 trials (3 si−6 s/12 s−3 sf). Premature responses were allowed and did not trigger timeout periods (for a description of the protocol, see Soares et al., 2018). Task acquisition was measured by the proportion of correct responses during training sessions. Moreover, two aspects of impulsive behavior were evaluated. Motor inhibition was assessed by the proportion of premature responses during the training protocol and both prematurity (PR) rate during the delays (amount of premature responses per minute of total delay), and the delay intolerance at the 3 sf trials after exposure to the longer intervals (PR rate at 3 sf/PR rate at 3 si) measures cognitive impulsivity. Auxiliary measures including latency to respond, to respond during each delay, and to collect rewards were also assessed.

#### 2.3.2. Probabilistic spatial reversal learning: behavioral flexibility

##### 2.3.2.1. Description of the apparatus

Rats were trained in the same six standard operant chambers described in the SIP procedure section but equipped with two retractable levers located on each side of the pellet dispenser and two lights above the levers.

##### 2.3.2.2. Behavioral procedure

We adapted the established serial PRL task (Alsiö et al., 2019) for levers. After 15 min of habituation to the test environment with free reward pellets in the pellet dispenser, animals were presented with the two levers illuminated. After the animal pressed either lever or after 30 s had passed since the lever presentation, the lever disappeared, and a pellet was delivered to the pellet dispenser. The rats earned a maximum of 100 pellets in this training session. If they did not complete all trials, the session terminated after 60 min. Subsequently, during Must Touch training, the rats had to press the illuminated lever for a reward. These sessions terminated following 60 min or after 100 rewards were earned, whichever occurred first. Next, the animals were trained to access the food storage using the pellet dispenser to begin a trial. This training phase was identical to Must Touch, except that all the animals had to emit an additional nose poke in the food dispenser to start each trial. These sessions also terminated following either 60 min or after 100 pellets earned. Finally, the rats were trained on a Punish Incorrect phase. This was identical to the previous Must Touch except that the presses on the non-illuminated lever were punished with a brief (5 s) timeout in complete darkness. Each training session was carried out for two consecutive days. The experimental sessions were conducted as the Punish Incorrect training, except that contingencies were modified so that one lever was randomly assigned a reward probability of 80% and the other a reward probability of 20%. Following eight consecutive correct responses (presses on the 80% reward-probability lever), the contingencies reversed so that the previous 20%-rewarded lever became 80%-rewarded and vice versa. The levers were presented for 30 s, and if there was no lever press within this period, the trial was deemed an omission, which triggered a 5-s timeout. The animals were given one session per day, each consisting of either 200 trials to be completed or 60 min. The learning criteria were more than three reversals completed per session, for three consecutive days. The main measures from the PSRL task were the number of sessions needed to achieve the criteria, the number of reversals completed per session, the win-stay probability (i.e., the probability to choose the same lever which was rewarded on the last trial), and the lose-shift probability (i.e., the probability to choose the alternative lever unrewarded on the last trial). Auxiliary measures including the proportion of correct and incorrect responses, accuracy, and latency to correct and incorrect responses and to collect rewards were also assessed.

#### 2.3.3. Rodent gambling task: risk decision-making

##### 2.3.3.1. Description of the apparatus

The animals were tested in six standard operant chambers identical to those described in the SIP procedure section with the array of five contiguous square holes opposite the pellet dispenser. All holes were active during the task except for the middle one.

##### 2.3.3.2. Behavioral procedure

The rats were habituated for 15 min to the test environment with free reward pellets in the pellet dispenser and in the response holes (except the center hole). Then, the rats were trained to make a nose poke into an illuminated response hole (1, 2, 4, 5) within 10 s to earn the reward. These sessions terminated following 30 min or after 100 rewards were earned, whichever occurred first. The criteria for progressing to the next training phase were the completion of 100 trials with ≥80% correct and ≤20% omitted. Next, the rats were trained on a forced-choice version of the rGT for seven sessions before the full free-choice task to ensure all the rats had equal experience with all four reinforcement contingencies and prevent potential biases toward a particular hole. Here, only one hole was illuminated. During experimental sessions, the animals started each trial by making a nose poke in the pellet dispenser. This response triggered the start of a 5-s intertrial interval (ITI). At the end of the ITI, holes 1, 2, 4, and 5 were illuminated for 10 s. An omission is scored if the rats failed to respond within 10 s and the animals could start a new trial with a nose poke in the pellet dispenser. A response in any illuminated hole turned off all stimulus lights and led to either delivery of reward or the start of a punishment period. If the trial was punished, no pellet was delivered and the stimulus light within the chosen hole flashed at 0.5 Hz until the punishment had finished. A nose poke in the pellet dispenser initiated the next trial after both reward and punishment. Premature responses during the ITI were punished by a 5-s timeout period, signaled by the illumination of the house light, after which the animals could start a new trial. Perseverative responses both after reward and during punishment were scored but not punished. The location of the pellet choice options (P1–4) was counterbalanced across rats such that half the animals were tested on version A and half on version B. The animals received six daily sessions per week until statistically stable patterns of choice behavior were observed over three sessions. Each session lasted for 30 min (for a description of the protocol, see Zeeb et al., 2009). The main measures from the rGT were the choice behavior (number of choices of a particular hole/total number of total choices); choice score [proportion of choice of the two advantageous options (P1 + P2), proportion of choice of the two disadvantageous options (P3 + P4)]; proportion of perseverative responses, proportion of perseverative responses during the punishment period (fraction of the total punishment duration); and proportion of perseverative responses after a reward was received (fraction of the total number of trials rewarded). Additional measures including latency to respond and to collect the reward were also assessed.

### 2.4. Statistical analysis

SIP acquisition was analyzed using a two-way repeated-measures analysis of variance (ANOVA), with “group” (LD and HD) as between-subject factor and “sessions” (20 sessions) as the within-subject factor. The analysis of VDS training was performed by two-way repeated-measures ANOVA with “group” (LD and HD) as between-subject factor and “sessions” (10 sessions) as the within-subject factor. PR rate and latency to respond during each delay were analyzed using repeated-measures ANOVA with “group” (LD and HD) as the between-subject factor and “delay” as the within-subject factor. Differences in delay tolerance and latency to respond and to collect reward were analyzed by Student's *t*-test (*t*-test). All variables in PSRL (total number of sessions needed to achieve the learning criteria, total number of reversals completed, win-stay and lose-shift probability, proportion of correct and incorrect responses, accuracy, latency to correct and incorrect response, and to collect the reward) were analyzed using a *t*-test. Regarding rGT, choice behavior was tested by a one-way ANOVA with “group” (LD and HD) as a between-subject factor, and the differences between groups in the remaining rGT variables (choice score, perseverative responses, perseverative responses during the punishment period, perseverative responses after a reward was received, and latency to respond and to collect the reward were also assessed) were analyzed by a *t*-test. The data expressed in percentages were arcsine transformed before analyses to limit the effect of an artificially imposed ceiling (McDonald, 2009). *Post-hoc* analyses were performed using Bonferroni correction when appropriate. Statistical significance was established at *p* < 0.05. Effect size is reported when appropriate; partial eta-squared values of 0.01, 0.06, and 0.14 and Cohen's *d* values of 0.2, 0.5, and 0.8 are considered to reflect small, medium, and large effects, respectively (Cohen, 1988). All analyses were performed with Statistica® software (version 8.0), and all figures were made using GraphPad Prism 8, except for the correlation matrices, carried out with JASP v0.13.

## 3. Results

### 3.1. Screening compulsivity by schedule-induced polydipsia

**Figure 2:**
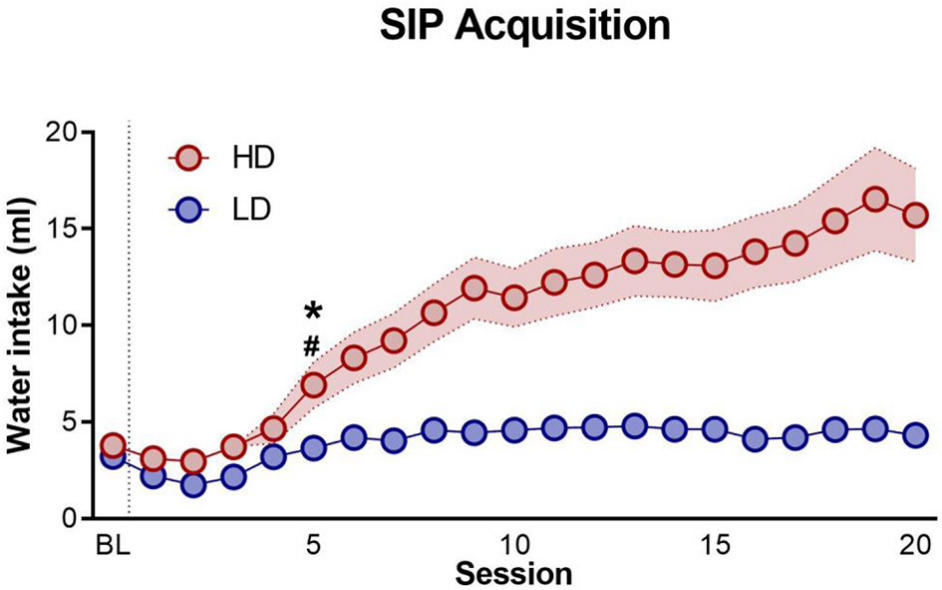
Schedule-induced polydipsia. The mean (± SEM) water intake in FT-60s across 20 sessions of schedule-induced polydipsia (SIP) in high-drinker (HD, *n* = 24) and low-drinker (LD, *n* = 24) rats. ^*^p < 0.05 indicates significant differences between HD and LD rats from that session onward. ^#^*p* < 0.05 indicates significant differences from that session onward compared with session 1 in the same group.

The mean water intake for LD and HD through 20 SIP sessions is shown in Figure 2. The mean total licks and total entries into food storage for LD and HD through 20 SIP sessions are not shown. The mean total number of water intake during the last 5 days of SIP was 4.37 ± 0.24 ml for LD and 15.14 ± 2.21 ml for HD. SIP acquisition was also evident in the total number of licks. The mean total number of licks during the last 5 days of SIP was 853.28 ± 93.82 for LD and 2934.75 ± 578.1 for HD. Repeated-measures ANOVA showed a significant interaction in water intake and LD vs. HD [interaction SIP session × group effect: *F*_(19,874)_ = 12.63, *p* < 0.001; η^2^*p* = 0.22]. Concerning the total number of licks, repeated-measures ANOVA revealed significant differences according to the interaction between the SIP acquisition sessions and LD vs. HD [interaction SIP session × group effect: *F*_(19,874)_ = 7.46, *p* < 0.001; η^2^*p* = 0.14]. *Post-hoc* analysis indicated that SIP induced different rates of drinking behavior across the 20 sessions in both groups. In water intake, the LD and HD groups differed in session 6 (*p* < 0.05; *d* = 0.84), and the HD group increased their number of licks in session 6 (*p* < 0.001; *d* = 1.11) compared with session 1. Similar differences between LD and HD were found in the total number of licks: the LD and HD group differed in session 5 (*p* < 0.05; *d* = 1.09), and the HD group increased their number of licks in session 5 (*p* < 0.05; *d* = 1.15) compared with session 1. There were no significant differences between LD and HD animals in the total entries into food storage on SIP [SIP session interaction × group effect: *F*_(19,874)_ = 1.27, *p* = 0.19].

### 3.2. Variable delay-to-signal

**Figure 3:**
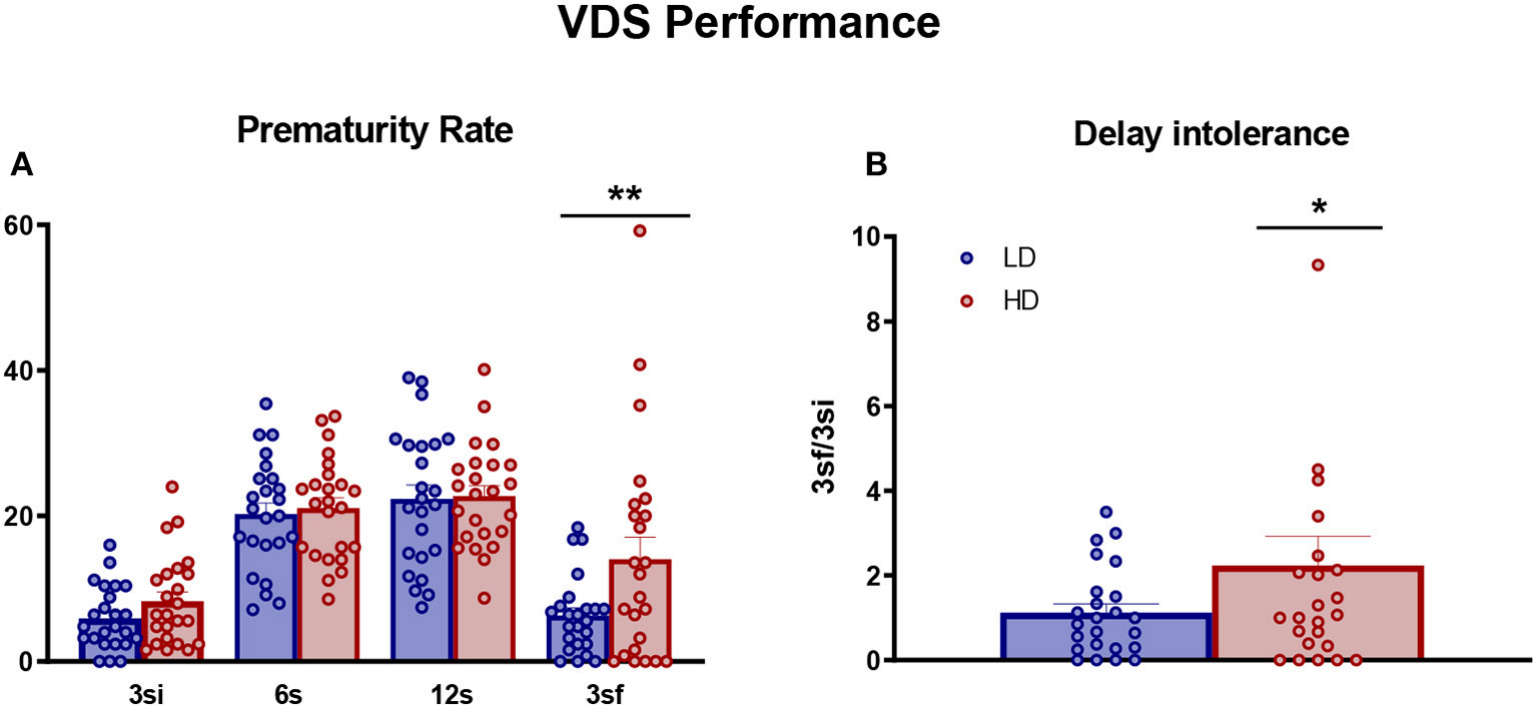
Variable delay-to-signal. The mean (± SEM prematurity rate **(A)** and delay intolerance (3s/3si) **(B)** on VDS task in high-drinker (HD, *n* = 24) and low-drinker (LD, *n* = 24) rats. *p < 0.05; **p < 0.01 indicate significant differences between HD and LD rats. 3sf, 3-s trials after exposure to the longer intervals; 3si, 3-s trials before exposure to the longer intervals.

Performance on VDS was measured in LD and HD animals and is shown in Figure 3. There were no significant differences between LD and HD animals during VDS training in the proportion of correct responses [data not shown. VDS session interaction × group effect: *F*_(9,414)_ = 0.99, *p* = 0.44] or in the proportion of premature responses [data not shown. VDS session interaction × group effect: *F*_(9,414)_ = 0.8, *p* = 0.61]. However, repeated-measures ANOVA showed a significant interaction in PR rate and LD vs. HD rats [Figure 3A; interaction VDS session × group effect: *F*_(3,138)_ = 2.94, *p* < 0.05; η^2^*p* = 0.06]. *Post-hoc* analysis indicated that HD animals presented a higher PR rate than LD counterparts at a 3 sf delay interval (*p* < 0.01; *d* = 0.69). This difference was also evident in the comparison between PR rate at 3 si and 3 sf delay intervals (Figure 3B; df = 46; *t*-test = −2.16; *p* < 0.04; *d* = 0.62). There were no differences between LD and HD rats in any auxiliary measures shown in Supplementary Table 1: latency to response (df = 46; *t*-test = 0.47; *p* = 0.64), latency to response in any delay [VDS delay interaction × group effect: *F*_(3,138)_ = 0.62, *p* = 0.6], and latency to collect reward (df = 46; *t*-test = 0.41; *p* = 0.68).

### 3.3. Probabilistic spatial reversal learning

**Figure 4:**
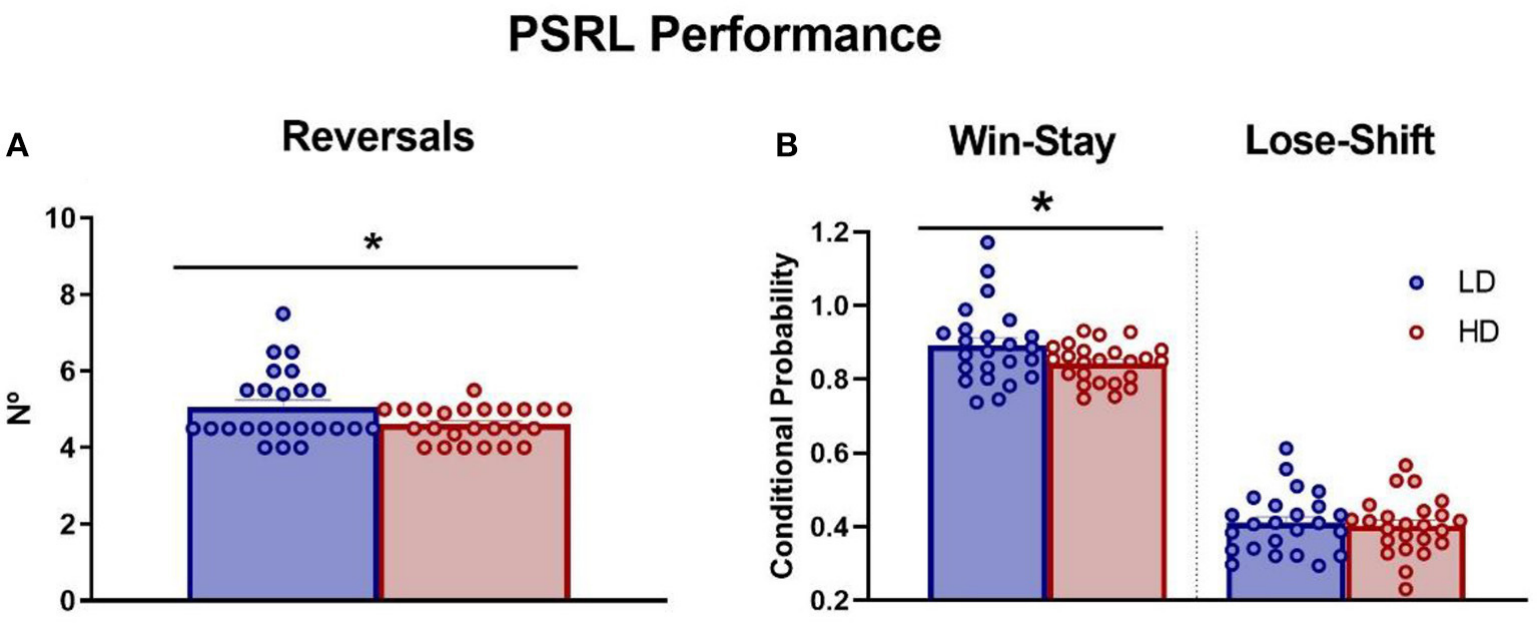
Probabilistic spatial reversal learning. The mean (± SEM) reversals completed per session **(A)** and win-stay/lose-shift conditional probability **(B)** on probabilistic spatial reversal learning in high-drinker (HD, *n* = 24) and low-drinker (LD, *n* = 24) rats. *p < 0.05 indicates significant differences between HD and LD rats.

The mean number of reversals completed, and the mean win-stay and lose-shift probability during the last three sessions were measured in LD and HD rats and are shown in Figure 4. The *t*-test analysis revealed that there were no differences in the number of sessions to achieve the criteria between groups (data not shown; df = 46; *t*-test = 1.2; *p* = 0.24), but interestingly, HD animals showed a smaller number of reversals completed compared with LD animals (Figure 4A; df = 46; *t*-test = 2.12; *p* < 0.05; *d* = 0.62). Concerning conditional probabilities, HD animals also showed decreased win-stay probability relative to LD animals (Figure 4B; df = 46; *t*-test = 2.03; *p* < 0.05; *d* = 0.63). No significant differences between the groups in lose-shift probability were found (Figure 4B; df = 46; *t*-test = 0.36; *p* = 0.72). There were no differences between LD and HD rats additional measures shown in Supplementary Table 2: proportion of correct responses (df = 46; *t*-test = 1.32; *p* = 0.19), proportion of incorrect responses (df = 46; *t*-test = −0.91; *p* = 0.36), accuracy (df = 46; *t*-test = 1.27; *p* = 0.21), latency to correct response (df = 46; *t*-test = −1.15; *p* = 0.26), latency to incorrect response (df = 46; *t*-test = −1.78; *p* = 0.08), or latency to collect the reward (df = 46; *t*-test = −0.06; *p* = 0.95).

### 3.4. Rodent gambling task

The choice score and each specific probability choice during the last three sessions to ensure the stability of the elections were measured. Under stable baseline performance across sessions 18, 19, and 20, there were no significant differences between LD and HD animals in the proportion of choice score [data not shown. rGT session interaction × group effect: *F*_(2,92)_ = 0.37, *p* = 0.69], p1 [data not shown. *F*_(2,92)_ = 0.35, *p* = 0.71], p2 [data not shown. *F*_(2,92)_ = 0.28, *p* = 0.75], p3 [data not shown. *F*_(2,92)_ = 0.35, *p* = 0.71], or p4 [data not shown. *F*_(2,92)_ = 0.47, *p* = 0.63]. However, only in p3, there was a significant group effect [*F*_(1,46)_ = 9.87, *p* < 0.01; η^2^*p* = 0.18].

**Figure 5:**
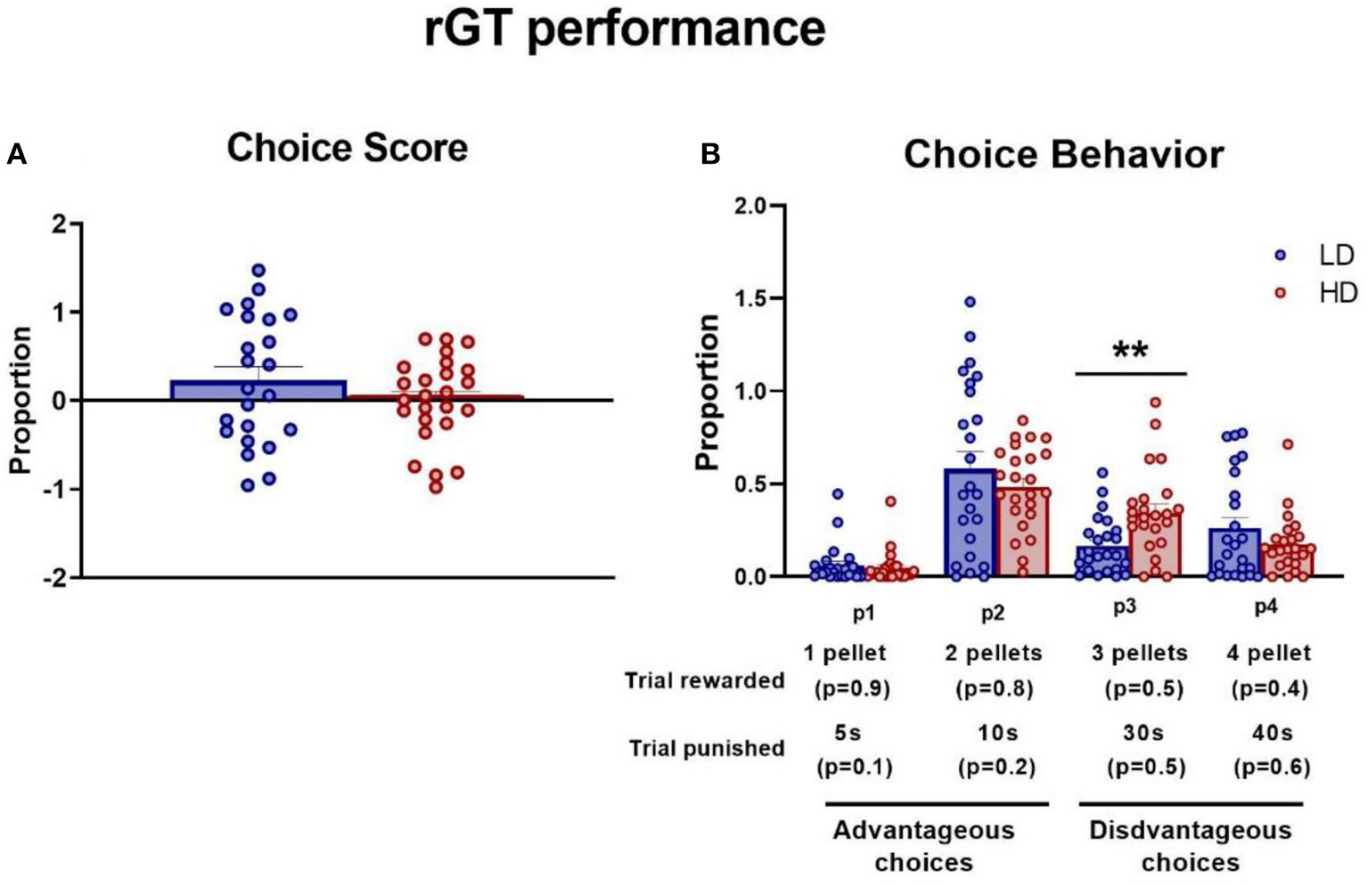
Rodent gambling task. The mean (± SEM) choice score **(A)** and choice behavior **(B)** on rodent gambling task (rGT) in high-drinker (HD, *n* = 24) and low-drinker (LD, *n* = 24) rats. **p < 0.01 indicates significant differences between HD and LD rats.

The mean (± SEM) proportion of choice score and choice behavior in each specific probability were assessed and are shown in Figure 5. The *t*-test did not reveal significant differences between groups in the choice score (Figure 5A; df = 46; *t*-test = 1.16; *p* = 0.25). However, ANOVA revealed significant differences between LD and HD in choice behavior [Figure 5B; *F*_(4,43)_ = 2.59, *p* < 0.05; η^2^*p* = 0.19]. *Post-hoc* analysis revealed that HD animals performed a higher proportion of p3 choices relative to LD animals (*p* < 0.01; *d* = 0.92).

**Figure 6:**
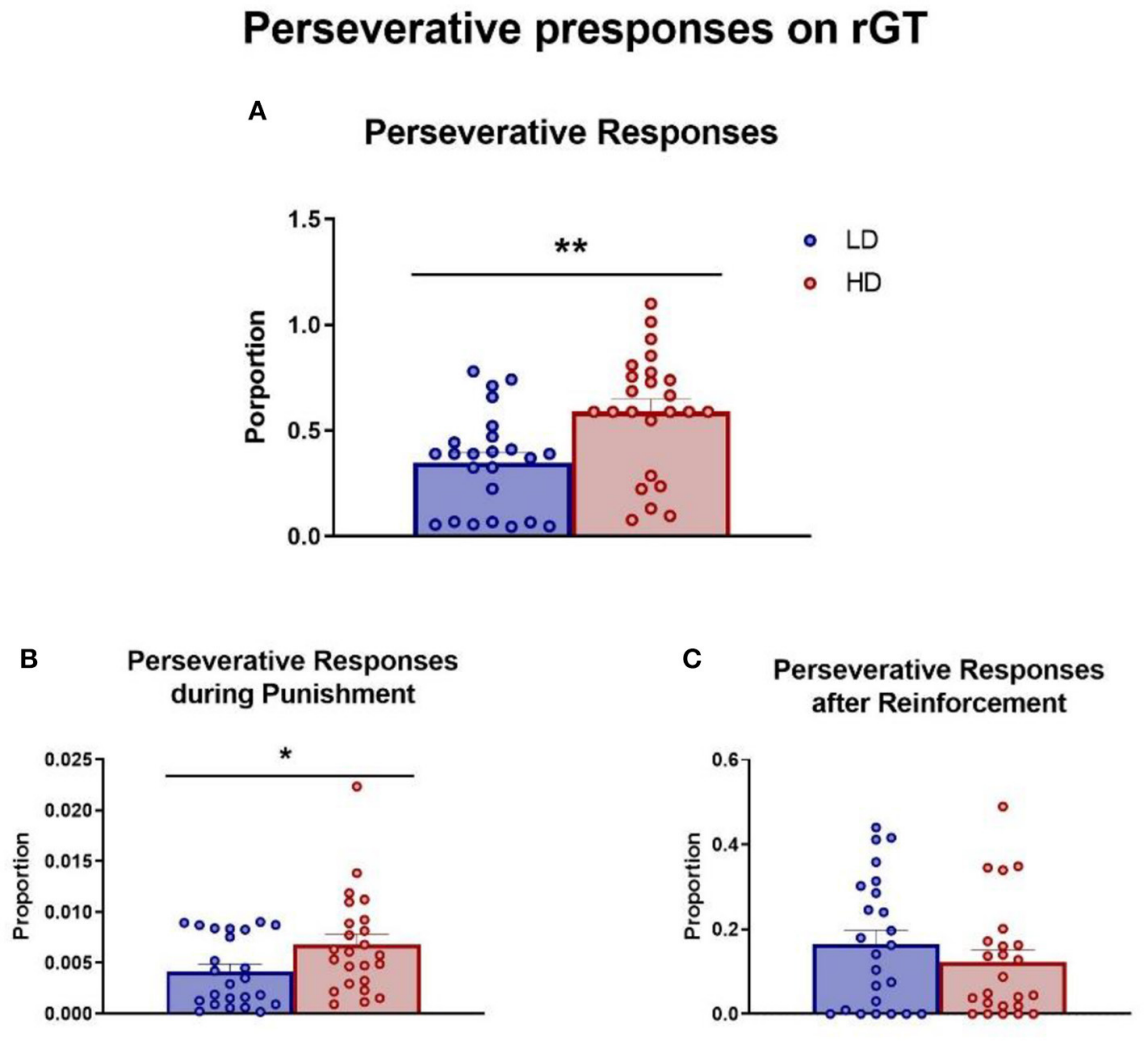
Rodent gambling task. The mean (± SEM) total perseverative responses **(A)**, perseverative responses during the punishment period **(B)**, and perseverative responses after a reward **(C)** on rodent gambling task (rGT) in high-drinker (HD, *n* = 24) and low-drinker (LD, *n* = 24) rats. *p < 0.05; **p < 0.01 indicate significant differences between HD and LD rats.

The mean (± SEM) proportion of total perseverative responses, perseverative responses during the punishment period, and perseverative responses after a reward were measured and are shown in Figure 6. The *t*-test analysis revealed that HD rats performed more total perseverative responses compared with LD rats (Figure 6A; df = 46; *t*-test = −3.25; *p* < 0.01; *d* = 0.94). Interestingly, this difference was also evident in the perseverative responses during the punishment period (Figure 6B; df = 46; *t*-test = −2.17; *p* < 0.05; *d* = 0.73), but there was no difference between groups in perseverative responses after a reward (Figure 6C; df = 46; *t*-test = 1.03; *p* = 0.31).

Finally, auxiliary measures are shown in Supplementary Table 3. There were no differences between HD and LD rats in either latency to respond (df = 46; *t*-test = 0.45; *p* = 0.65) or latency to collect reward (df = 46; *t*-test = −0.11; *p* = 0.91).

### 3.5. Correlation matrix between behavioral outcomes

**Figure 7:**
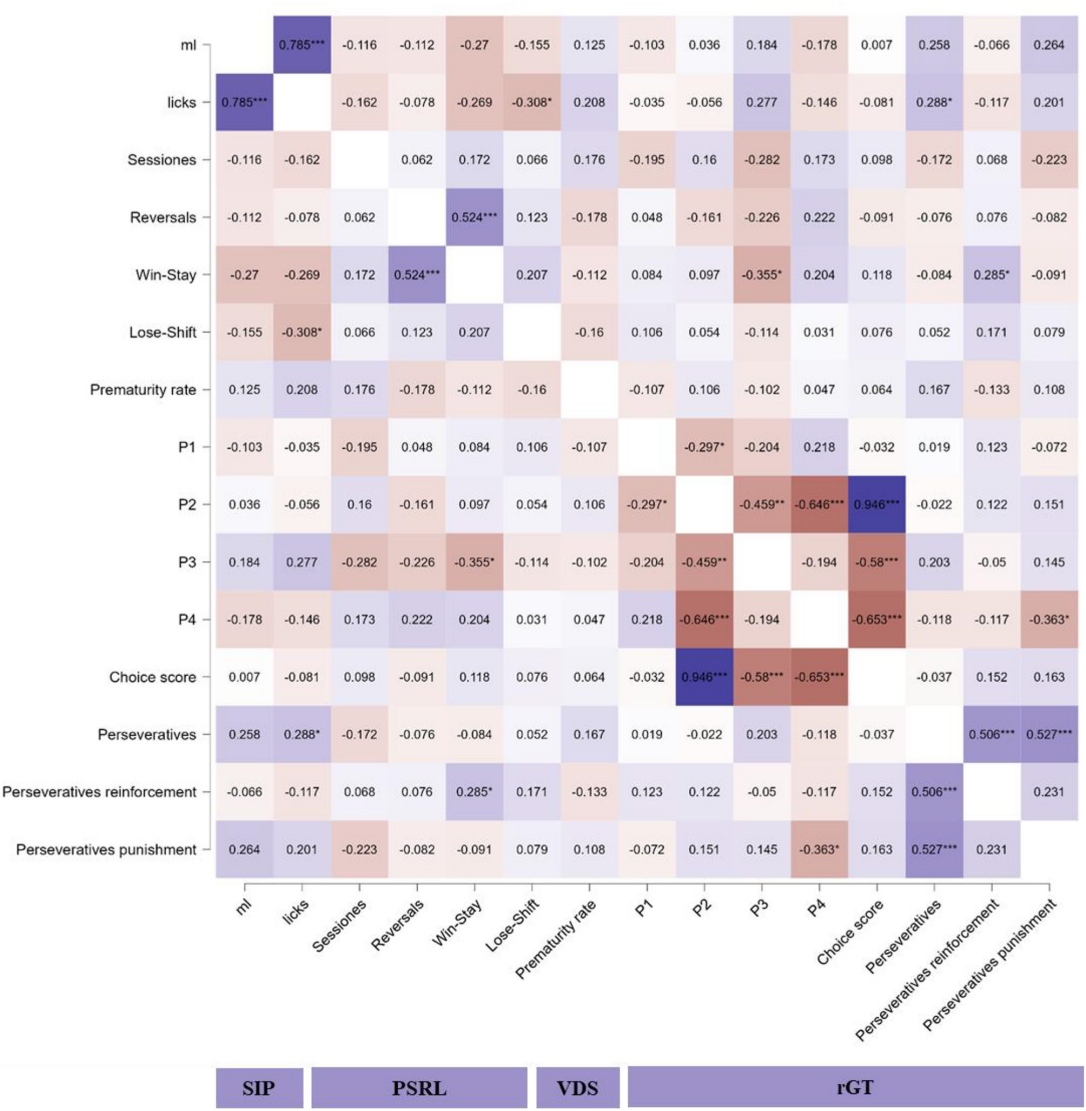
Heatmap of correlations between the main measures on SIP, PSRL, VDS, and rGT. PSRL: probabilistic spatial reversal learning; rGT: rodent gambling task; SIP: schedule-induced polydipsia; VDS: variable delay-to-signal. *p < 0.05; **p < 0.01; ***p < 0.001 indicate significant correlations between variables.

The correlation matrix between different behavioral measures on SIP, PSRL, VDS, and rGT is shown in Figure 7. Significant positive high correlations were found between water intake (ml) and licks to the bottle on SIP (r = 0.785; *p* < 0.001) and between choice score and choices to P2 on rGT (*r* = 0.946; *p* < 0.001). Significant positive medium correlations were found between win-stay probability and reversal completed on PSRL (*r* = 0.524; *p* < 0.001), total perseverative responses on rGT and both, perseverative responses during punishment (*r* = 0.527; *p* < 0.001), and reinforcement on rGT (*r* = 0.527; *p* < 0.001). Significant negative medium correlations were found between licks to the bottle on SIP and lose-shift probability on PSRL (*r* = −0.308; *p* = 0.03), win-stay probability on PSRL, and choices to P3 on rGT (*r* = −0.355; *p* = 0.01), choices to P2 and P3 on rGT (*r* = −0.459; *p* = 0.001), choices to P2 and P4 on rGT (*r* = −0.646; *p* < 0.001), choices score and both, choices to P3 (*r* = −0.58; *p* < 0.001), and P4 (*r* = −0.653; *p* < 0.001) on rGT, and perseverative responses during punishment and choices to P4 (*r* = −0.363; *p* = 0.01).

## 4. Discussion

In the present study, we demonstrated that compulsive HD animals exhibited increased cognitive impulsivity by delay intolerance on the VDS task, behavioral inflexibility by a reduced number of reversals on the PSRL, with less sensitivity to positive feedback demonstrated by a decreased win-stay strategy, and higher cognitive impulsivity by risky decision-making on the rGT, relative to LD animals. However, HD did not differ from LD rats in motor inhibition on the VDS. The differences between HD and LD rats observed in these tasks might not be attributable to possible neurobehavioral changes induced by SIP. Previous studies from our laboratory have shown differences between HD and LD in traits related to compulsive phenotypes, such as cognitive inflexibility, before SIP acquisition (Merchán et al., 2019). It points toward presumably preexisting differences in HD rats regarding the vulnerable trait of compulsivity. Moreover, the assessment of volumetric brain changes in HD and LD rats revealed no differences 1 month after SIP (Mora et al., 2020). Therefore, taking into account that there were 20 days of resting time between each behavioral task, we might discard possible interferences of SIP and between each of the behavioral tasks used on the behavioral differences observed. These results are discussed in terms of the dissociable contribution of different neurocognitive and neurobehavioral domains in the compulsivity phenotype.

### 4.1. Increased cognitive impulsivity in compulsive HD rats selected by SIP

The assessment of motor and cognitive impulsivity by VDS revealed that compulsive HD animals selected by SIP presented increased cognitive impulsivity in terms of delay intolerance, compared with LD animals. However, no differences were observed between groups in motor inhibition measure, nor in learning during task acquisition training sessions. On the one hand, compulsive HD rats exhibited delay intolerance after exposure to long delay periods, showing increased premature responses at 3 sf delay trials relative to LD rats. These results are in accordance with previous studies that have shown increased impulsive choice on a DDT in rats with compulsive drinking behavior on SIP (Cardona et al., 2006, 2011; Ibias and Pellón, 2011). The link between compulsive behavior and rigid choice pattern has also been shown by a novel animal model of ADHD (Leo et al., 2018); thus, when a strain of dopamine transporter (DAT)-knockout (KO) was compared with DAT heterozygous (HET) and wild-type (WT) rats on an intolerance-to-delay task (IDT), KO rats reacted to the increasing delay with motor stereotypies such as sniffing or chewing the feeding storage area (Cinque et al., 2018). In clinical studies, OCD patients also show higher cognitive impulsivity than healthy controls (Benatti et al., 2014; Pinto et al., 2014; Sohn et al., 2014; Grassi et al., 2018, 2020). On the other hand, the lack of differences between the groups in motor inhibition is in accordance with previous data from our group, where only a trend to increase premature responses was found in HD compared with LD rats on the 5-CSRTT (Moreno et al., 2010). A possible explanation for the lack of a motor inhibition deficit in the compulsive phenotype of HD rats selected by SIP might be due to its observation in basal conditions, but when environmental demands increase, HD rats become more vulnerable to developing a deficit in motor inhibition. In this sense, HD animals presented a greater vulnerability to motor disinhibition compared with LD rats, observed by a leftward shift in the premature responses induced by the dose–response effect to D-amphetamine on the 5-CSRTT (Moreno et al., 2010). Moreover, motor impulsivity is found to increase in OCD patients (Chamberlain, 2006; Chamberlain et al., 2007; Morein-Zamir et al., 2010; Boisseau et al., 2012; Sohn et al., 2014), and the ability to resist immediate reinforcement opportunities under distress is an important key to the inhibition of intrusions in OCD patients (Macatee et al., 2016).

### 4.2. Increased behavioral inflexibility and decreased positive feedback sensitivity in compulsive HD rats selected by SIP

Regarding behavioral flexibility measurement, HD compulsive rats selected by SIP showed increased behavioral inflexibility on PSRL compared with LD rats measured by the reduced number of reversals completed during the last three sessions. It is important to mention that these differences are not due to a learning deficit, as both groups of rats needed the same number of sessions to achieve behavioral stability criteria (more than three reversals completed per session for three consecutive days). This failure in the ability to adapt the behavior to a changing environment is in accordance with previous data found from our laboratory, where compulsive HD rats selected by SIP showed behavioral inflexibility by an increased number of perseverative errors and trials to complete the criterion in reversal sessions on other models of spatial-discrimination reversal learning tasks such as serial reversal learning (Navarro et al., 2017) and within-session reversal learning (Merchán et al., 2019) and in the increased latency to find the platform in the reversal sessions on the Morris water maze (Prados-Pardo et al., under review). An alteration in the serotonergic 5-HT system has been proposed as a potential underlying mechanism related to the behavioral inflexibility observed in compulsive HD rats selected by SIP. Thus, converging evidence has shown that compulsive HD rats selected by SIP and by their inflexible behavior on reversal learning both have a deficit in the serotonin 5-HT2A receptor in the FC (Moreno et al., 2010; Barlow et al., 2015; Mora et al., 2018). Moreover, increasing 5-HT function by citalopram administration improved PSRL performance increasing the number of reversals completed (Bari et al., 2010) and reduced compulsive drinking behavior on SIP (Navarro et al., 2015) also showed using DOI, a 5HT2A/C selective agonist (Mora et al., 2018). Reducing serotonin activity by chronic 5-HT depletion also impaired reversal performance (Clarke et al., 2004, 2007; Bari et al., 2010) and increased compulsive drinking behavior (Merchán et al., 2017). These cognitive inflexibility data on the different protocols of reversal learning might be understood in terms of an imbalance in the frustrative non-reward construct, as they are associated with the reactions elicited in response to withdrawal/prevention of reward (Blanchard et al., 2011).

Moreover, the reversal learning protocol used in the present study expands the knowledge about the behavioral inflexibility deficit in the compulsive HD rats selected by SIP, as the results point toward an alteration in processing the win-stay strategy on the PSRL, linking the cognitive control system with the negative valence system. Compulsive HD animals exhibited decreased sensitivity to positive feedback compared with LD animals, showing decreased conditional win-stay probability (i.e., the probability of pressing the same lever rewarded in the previous trial). The insensitivity to reward contingencies has also been shown in different preclinical models of compulsive-like behavior: first, compulsive HD rats selected by SIP exhibited insensitivity to reinforcer devaluation and excessive habit formation measured by similar lever pressing under extinction after the consumption of either a different reinforcer or the same reinforcer compared with LD rats (Merchán et al., 2019); second, the rats exposed to chronic intermittent voluntary alcohol consumption, a model of alcohol use disorder, used win-stay strategy less than H^2^O-drinking rats (Aguirre et al., 2020); third, the animals exposed to alternation of a standard chow with a high palatable diet, a model of compulsive eating behavior, showed reduced sensitivity to d-amphetamine, suggestive of a hypofunctional reward system (Moore et al., 2020); and fourth, transgenic SAPAP3^−/−^ mice showed lower response rates and fewer attempts to collect food pellets in schedules of reinforcement tasks than wild-type mice, indicating altered reward processing (Ehmer et al., 2020). Finally, mirtazapine, an antidepressant with specific serotonergic effects, significantly increased the sensitivity to positive feedback, increasing the proportion of the win-stay strategy on the PSRL (Drozd et al., 2019). Therefore, the 5-HT modulation seems to be crucial in behavioral flexibility strategies to remediate compulsive behaviors. In clinical studies, cognitive flexibility impairment has been also observed in OCD patients and their unaffected relatives (Chamberlain, 2006; Chamberlain et al., 2007; Patel et al., 2010; Voon et al., 2015; Vaghi et al., 2017) and also in patients with other obsessive-compulsive spectrum disorders such as obsessive-compulsive personality disorder (Fineberg et al., 2015) and schizophrenia with comorbidity with OCD (Patel et al., 2010). Interestingly, there may be a relationship between behavioral inflexibility and reduced positive feedback sensitivity present in HD rats and the dopaminergic mechanisms of compulsive drug taking. Indeed, the use of agents that enhance dopamine signaling reduced the compulsive water intake on SIP, and HD and LD differ in their dopamine binding to D1 and D2 receptors in mesolimbic areas (for a review see Martín-González et al., 2022a). Furthermore, preclinical and clinical literature describes that the predisposition to develop addiction behavior might be linked to a loss of functional autonomy of the dopamine mesolimbic seeking/exploration system (Alcaro et al., 2021).

### 4.3. Increased impulsive risky decision-making in compulsive HD rats selected by SIP

The measurement of risky decision-making on the rGT revealed that compulsive HD animals selected by SIP showed a higher proportion of choices of a hole associated with a disadvantageous probability p3 (probability to earn e pellets = 0.5; probability to receive 30 s of punishment = 0.5) compared with LD rats, although there were no differences between the groups in the choice score. There were no significant differences between the groups in learning performance, which supports the notion that these differences were not due to a deficit in acquisition, retention, or food motivation. Preclinical studies on inhibitory control deficit using traumatic brain injury (TBI), which replicate impulse control and decision-making impairment observed in humans, showed that TBI animals presented chronic vulnerability to risk-based decision-making in the rGT, showing a preference for the riskiest and most suboptimal option over all others (Shaver et al., 2019; Ozga-Hess et al., 2020). Moreover, 5-HT might also play a key role in the modulation of risk-taking behavior. Intra-lOFC infusions of the 5-HT2C antagonist RS 102221 reduced risky choice in animals that showed a preference for the risky options of the rGT at baseline (Hathaway et al., 2021), and also the 5-HT 2C receptor blockade by SB 242,084 administration improves decision-making when rewards are paired with audiovisual cues in a rat gambling task (Adams et al., 2017). Clinical literature reveals that OCD patients tend to make risky decisions, favoring options that provide large initial rewards but ultimately lead to a disadvantageous outcome (Cavedini et al., 2002, 2010, 2012; da Rocha et al., 2008, 2011; Kodaira et al., 2012; Grassi et al., 2015, 2018, 2020; Kim et al., 2015; Zhang et al., 2015).

Moreover, HD animals performed more perseverative responses during the sessions, specifically during the punishment period. These data suggest a possible relationship between compulsive drinking behavior on SIP and other compulsive behaviors such as a greater propensity to enhanced perseverative responses under extinction conditions on 5-CSRT task (Moreno et al., 2010), elevated compulsive lever pressing during the pre-training phase of latent inhibition paradigm (Navarro et al., 2017), and a higher number of marbles partially buried on the marble burying test (Prados-Pardo et al., 2019). The fact that differences between groups in perseverative responses were evident during the punishment period might be related to the compulsive behavior function of avoiding perceived negative consequences (Fineberg et al., 2014; Banca et al., 2015). The relation between compulsivity and avoidance is in accordance with previous results in our group: first, HD animals were more resistant to fear extinction on the PA test, shown by a sustained higher latency to enter the dark compartment at the last extinction session, 10 days after receiving an electric shock, compared with LD rats (Martín-González et al., 2022b); second, Roman high-avoidance (RHA) rats, selected by their avoidance performance in the active avoidance (AA) test, showed compulsive drinking on SIP (Moreno et al., 2010), with a longer time to lead the extinction in cocaine self-administration procedure (Fattore et al., 2009), and the partial reinforcement extinction effect on Pavlovian autoshaping procedure was larger and longer lasting in RHA (Fuentes-Verdugo et al., 2020) than in Roman low avoidance (RLA). Thus, a classic explanation of this phenomenon is that excessive drinking may be a coping response to stress caused by intermittent food delivery (Brett and Levine, 1979, 1981; Wallace et al., 1983; Tazi et al., 1986; Dantzer et al., 1988; Mittleman et al., 1988; López-Grancha et al., 2006; Martín-González et al., 2022b) and might be modulated by HPA axis (Fuentes et al., 2014; Merchán et al., 2019; Martín-González et al., 2022b). Avoidance and perseverative responses point toward a dysfunctional processing of explicit contingencies that have been proposed to be undermined in compulsive disorders (Fineberg et al., 2018) and highlight the overlap of the cognitive control system and the negative valence system.

### 4.4. Is there any relationship between behavioral variables in compulsive HD rats?

As shown in the correlation matrix, there were some relationships between measures on the same tasks, such as water intake and licks to the bottle on SIP, showing that both are assessing the same variable, in this case, compulsive behavior. That is the case of the correlation between perseverative responses and perseverative responses during reinforcement or punishment on rGT, showing an inhibition deficit on this task. Moreover, on PSRL there might be a relationship between win-stay probability and the number of reversals completed per session, showing that sensitivity to positive feedback is necessary for performing this task properly. Regarding rGT, a clear relationship between choices to P2 (an optimal option) and the choice score was found, suggesting that this hole might be the most determinant for achieving high accuracy on the task. Indeed, the suboptimal options (P3 and P4) negatively correlated with the choice score, and choices to P2 correlated negatively with choices to both, P3 and P4, pointing toward this idea. Finally, there might be relationships between variables of different tasks, such as a negative correlation between compulsive licking behavior on SIP and the sensitivity of negative consequences on PSRL, that might show the perseveration of compulsive behavior, which leads to following rigid strategies, despite negative consequences (Chamberlain et al., 2009; Robbins and Crockett, 2010). It seems that compulsive HD animals develop an aberrant behavior when faced with negative consequences, being insensitive to reinforcement. Thus, the persistence of rigid and habitual compulsive responses, which constitute a failure of flexibility, update the reward, safety, or harm signals, might point to the relationship between cognitive control and negative valence systems. Moreover, the effects of exposure to uncertainty conditions on VDS, PSRL, and rGT tasks promote a risky and rigid decision-making strategy (Fugariu et al., 2020). In this sense, behavioral inflexibility might be acting as a modulator of other behavioral impairments (Hathaway et al., 2021) by enhancing cognitive impulsivity in terms of delay intolerance and risky decision-making.

Finally, the present study shows some limitations. First, an increase in the number of animals per group using larger sample sizes might help to extract more robust statistical differences in the behavioral measures on the different tests used, as well as to perform a more informative correlation analysis. Second, the correlation analysis performed shows a potential relationship between the behavioral domains assessed. However, further studies might analyze the microstructural differences in some types of behaviors, disentangling whether the differences between groups depend on how LD and HD learn to seek or to avoid negative contingencies, highlighting the relationship between cognitive control and negative valence system domains on the development of compulsive behaviors (Moreno-Montoya et al., 2022). Indeed, increasing the sample size would also help to improve the assessment and use of an integrative statistical approach across all tasks. Finally, in the present study, we did not investigate sex differences in these behavioral measures, since, as the clinical literature shows, the prevalence of compulsive spectrum disorders is higher in male patients. However, future research should carry out studies in order to clarify the possible sexual dimorphism.

In summary, the present study suggests the relevance of the characterization of different constructs related to the compulsive phenotype as those linked not only to cognitive control but also to the negative valence system domain. Thus, according to the results, the compulsive HD rats selected by SIP have been characterized by impairments in cognitive impulsivity in terms of delay intolerance, behavioral inflexibility with insensitivity to positive feedback, and risky decision-making with perseverative responses under punishment periods. However, more research in the neurobehavioral mechanisms involved in the cognitive, emotional, and behavioral patterns of response in the compulsive phenotype would improve the knowledge of this transdiagnostic trait, for generating better diagnoses, treatments, and prevention strategies.

## Data availability statement

The raw data supporting the conclusions of this article will be made available by the authors, without undue reservation.

## Ethics statement

All the procedures were approved by the Committee of Ethics of the University of Almería and by the Junta de Andalucía and were carried out in accordance with the Spanish Royal Decree 53/2013 and the European Community Directive (2010/63/EU) for animal research.

## Author contributions

EM-G, SM, and MM-M contributed to the conception and design of the study. EM-G, ÁP-P, and MO-C collected and analyzed the data. DC-G contributed with methodological assistance. PF contributed to the data interpretation. EM-G wrote the first draft of the manuscript. SM and MM-M supervised all the experimental processes. MM-M also assisted with the resources, the project administration, and the funding acquisition. All authors contributed to the manuscript revision, read, and approved the submitted version.
