# Supplementary material for: Behavioral domains in compulsive rats: implications for understanding compulsive spectrum disorders
Source: Front Behav Neurosci. 2023 May 18;17:1175137. doi: 10.3389/fnbeh.2023.1175137 (PMC10234153; doi:10.3389/fnbeh.2023.1175137)
Supplement: Supplementary file 1 [file Table_1.DOCX]

Supplementary Material

Behavioral Domains in Compulsive Rats: Implications for Understanding Compulsive Spectrum Disorders.

Elena Martín-González*, Manuela Olmedo-Córdoba, Ángeles Prados-Pardo, Daniel J. Cruz-Garzón, Pilar Flores, Santiago Mora, Margarita Moreno-Montoya.

*** Correspondence:** Margarita Moreno: [mgmoreno@ual.es](mailto:mgmoreno@ual.es)

# Supplementary Tables

|  | **LD** | **HD** |
| --- | --- | --- |
| Latency to response (s) | 48.96 ± 1.29 | 48.06 ± 1.39 |
| Latency to response delay 3si (s) | 8.13 ± 0.8 | 6.95 ± 0.55 |
| Latency to response delay 6s (s) | 14.9 ± 0.94 | 14.86 ± 1.55 |
| Latency to response delay 12s (s) | 16.16 ± 0.92 | 17.06 ± 1.32 |
| Latency to response delay 3sf (s) | 9.46 ± 0.8 | 8.52 ± 0.56 |
| Latency to collect reward (s) | 11.58 ± 0.61 | 11.16 ± 0.81 |

**Supplementary Table 1.** Auxiliary measures on Variable delay-to-signal. Data are expressed as the means ± SEM.

|  | **LD** | **HD** |
| --- | --- | --- |
| Correct Responses (Proportion) | 0.69 ± 0.01 | 0.69 ± 0.01 |
| Incorrect Responses (Proportion) | 0.37 ± 0.01 | 0.38 ± 0.01 |
| Accuracy | 0.69 ± 0.01 | 0.68 ± 0.01 |
| Latency to correct response (s) | 903.42 ± 46.35 | 987.36 ± 56.57 |
| Latency to incorrect response (s) | 880.21 ± 43.32 | 1015.54 ± 62.23 |
| Latency to collect reward | 13.84 ± 2.17 | 14.02 ± 2.29 |

**Supplementary Table 2.** Auxiliary measures on Probabilistic Spatial Reversal Learning. Data are expressed as the means ± SEM.

|  | **LD** | **HD** |
| --- | --- | --- |
| Latency to response (s) | 577.55 ± 56.33 | 542.06 ± 54.21 |
| Latency to collect reward (s) | 24.22 ± 3.07 | 24.67 ± 2.72 |

**Supplementary Table 3.** Auxiliary measures on Rodent Gambling Task. Data are expressed as the means ± SEM.
